# Understanding the Clinical Use of Levosimendan and Perspectives on its Future in Oncology

**DOI:** 10.3390/biom13091296

**Published:** 2023-08-24

**Authors:** Eduarda Ribeiro, Nuno Vale

**Affiliations:** 1 OncoPharma Research Group, Center for Health Technology and Services Research (CINTESIS), Rua Doutor Plácido da Costa, 4200-450 Porto, Portugal; eduardaprr@gmail.com; 2 CINTESIS@RISE, Faculty of Medicine, University of Porto, Alameda Professor Hernâni Monteiro, 4200-319 Porto, Portugal; 3 Institute of Biomedical Sciences Abel Salazar (ICBAS), University of Porto, Rua de Jorge Viterbo Ferreira 228, 4050-313 Porto, Portugal; 4 Department of Community Medicine, Health Information and Decision (MEDCIDS), Faculty of Medicine, University of Porto, Rua Doutor Plácido da Costa, 4200-450 Porto, Portugal

**Keywords:** levosimendan, cancer, drug repurposing, drug combinations, vasodilatory mechanisms

## Abstract

Drug repurposing, also known as repositioning or reprofiling, has emerged as a promising strategy to accelerate drug discovery and development. This approach involves identifying new medical indications for existing approved drugs, harnessing the extensive knowledge of their bioavailability, pharmacokinetics, safety and efficacy. Levosimendan, a calcium sensitizer initially approved for heart failure, has been repurposed for oncology due to its multifaceted pharmacodynamics, including phosphodiesterase 3 inhibition, nitric oxide production and reduction of reactive oxygen species. Studies have demonstrated that levosimendan inhibits cancer cell migration and sensitizes hypoxic cells to radiation. Moreover, it exerts organ-protective effects by activating mitochondrial potassium channels. Combining levosimendan with traditional anticancer agents such as 5-fluorouracil (5-FU) has shown a synergistic effect in bladder cancer cells, highlighting its potential as a novel therapeutic approach. This drug repurposing strategy offers a cost-effective and time-efficient solution for developing new treatments, ultimately contributing to the advancement of cancer therapeutics and improved outcomes for patients. Further investigations and clinical trials are warranted to validate the effectiveness of levosimendan in oncology and explore its potential benefits in a clinical setting.

## 1. Introduction to Drug Repurposing

The process of developing new drugs is time-consuming, challenging and expensive. However, an alternative and effective strategy called drug repurposing offers a promising approach by utilizing existing approved drugs for new medical indications [1]. This approach not only reduces the costs and time required for development but also provides alternative clinical options [2,3].

Drug repurposing, also known as repositioning, reprofiling or re-tasking, revolves around the concept of discovering novel applications for drugs that have already gained approval [4]. The key to drug repurposing lies in the available knowledge about the bioavailability, pharmacokinetics, dosage, safety, efficacy and toxicity of these approved drugs [5]. By leveraging this existing data, researchers can save time and resources, opening doors to the development of low-cost therapies for various diseases, including cancer. In essence, drug repurposing accelerates the implementation of new treatments and the discovery of new targets, making it a cost-effective and time-efficient approach.

In the past, drug repurposing often occurred serendipitously, where the unexpected effects of a drug led to further investigations and new applications. For example, zidovudine, which was originally developed for cancer, subsequently earned the distinction of being the first medication approved by the FDA for HIV treatment [6]. Minoxidil, which was intended for hypertension, was discovered to have hair growth effects through retrospective clinical analysis [7].

Doxorubicin belongs to anthracycline antibiotics approved for medical use in the United States in the late 1950s [8]. Thanks to its efficacy and broad effect against a variety of cancers, in 1974 this drug was approved by the FDA as an effective anticancer drug [9].

Another example of successful drug repurposing is thalidomide for the treatment of multiple myeloma. Thalidomide, initially conceived as a sedative, gained popularity among pregnant women due to its ability to treat nausea in pregnancy. However, its use was later discovered to cause severe congenital malformations, leading to its withdrawal from the market [10]. In 2006, the use of thalidomide was approved in combination with the steroid dexamethasone for the treatment of multiple myeloma [11].

Remarkably, phosphodiesterase 5 (PDE5) inhibitors such as sildenafil were initially developed to treat angina, a disorder characterized by reduced blood flow to the heart. Later, this drug was found to have beneficial effects on erectile dysfunction, leading to their wide use in this context. This successful drug repurposing raises the possibility that PDE5 inhibitors could have additional therapeutic applications beyond their original intended use. With this, sildenafil has also emerged as a potential anticancer agent, considering the urgent need for new cancer therapies [12,13,14,15,16,17,18].

Drug repurposing faces challenges related to intellectual property and economic viability because in certain countries, obtaining patents for additional medical uses is impeded by legal obstacles, which undermines the motivation for pursuing drug repurposing efforts. Data access and compound availability, and the reluctance of pharmaceutical companies to share their compound libraries, hampers the possibilities for drug repurposing, and there is the potential saturation of opportunities for a given disease. However, innovative approaches such as exploring new targets, studying drug combinations and leveraging precision/systems medicine can mitigate these challenges and continue expanding the scope of drug repurposing [19].

Moreover, the use of well-established drugs for purposes beyond their original intended use, such as vasodilators, is a new strategy for treating cancer. In a previous study [20], our group hypothesized that the use of levosimendan could enhance the cytotoxic effect of 5-fluorouracil (5-FU) in bladder cancer cells. The rationale behind this strategy is grounded in the fact that the toxicological profiles of the repurposed drugs are already well-documented and deemed safe. By investigating their potential anticancer effects more comprehensively, we can unlock opportunities to incorporate these drugs into clinical practice with a higher level of confidence. By identifying a suitable repurposed drug that complements the antitumor activity of 5-FU we hope to create a synergistic effect, thereby amplifying the therapeutic benefits. This approach not only offers a safe starting point with an established antineoplastic drug, but also opens up new possibilities for combination therapies that could lead to improved treatment outcomes for patients with cancer. The knowledge of levosimendan’s toxicological characteristics adds an extra layer of assurance, increasing the likelihood of successful translation from preclinical research to clinical applications. This drug repurposing strategy holds significant promise in advancing cancer therapeutics, as it capitalizes on existing drug safety profiles while exploring their potential anticancer properties more deeply. Ultimately, our aim is to contribute to the development of more effective and safer treatments for cancer patients.

The repurposing of this drug to treat cancer has never been studied before. Thus, we are the first group to present the potential antitumour mechanisms of this drug.

In summary, the strategy of drug repurposing presents a simplified and cost-effective way to uncover new medical applications for drugs that are already in existence. By capitalizing on the knowledge and data already available for approved drugs, researchers can expedite the development of low-cost therapies and facilitate a new target discovery.

## 2. The Drug Levosimendan

Levosimendan [the (−) enantiomer of 4-(1,4,5,6-tetrahydro-4-methyl-6-oxo-3-pyridazinyl)phenylhydrazonopropanedinitrile] is a calcium sensitizer that belongs to a pyridazinone-dinitrile derivative molecule [21]. The year 2000 marked the attainment of regulatory approval for this drug’s broad utilization, specifically aimed at addressing the decompensation of severe chronic heart failure [22].

### 2.1. Pharmacodynamics

Levosimendan exerts its positive inotropic effects through a mechanism called calcium sensitization that binds to cardiac troponin C (cTnC) in a calcium-dependent manner, thus stabilizing the troponin complex. This interaction enhances the sensitivity of cardiac muscle cells to the available calcium ions [23]. By stabilizing troponin C, levosimendan promotes actin–myosin cross-bridging, leading to increased cardiac contractility without a concurrent increase in myocardial consumption of adenosine triphosphate (ATP). This means that the drug improves cardiac performance and contractility without significantly increasing the overall energy demand and oxygen consumption of the myocardium [21,24].

One important benefit of the levosimendan mechanism of action is that it does not raise the total intracellular calcium levels, which can contribute to arrhythmias. By avoiding an excessive increase in intracellular calcium, levosimendan has a reduced potential to cause arrhythmias compared to other positive inotropic agents [25].

### 2.2. Pharmacokinetics

**Figure 1:**
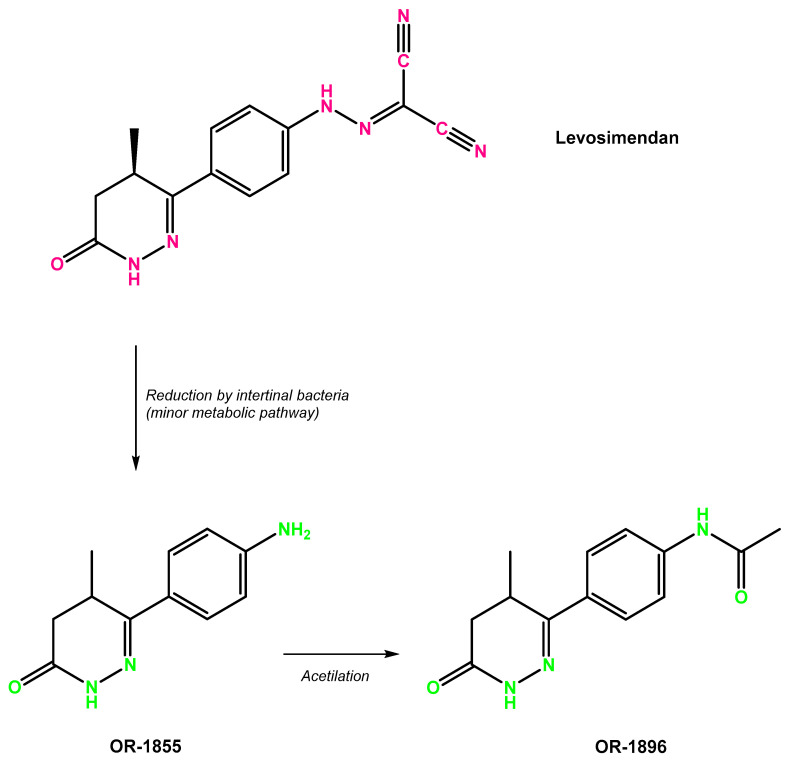
Metabolic pathway of transformation of levosimendan in its active metabolites.

Levosimendan exhibits linear pharmacokinetics within the therapeutic dose range (0.05 to 0.2 μg/kg/min) [26]. Within around 60 min of intravenous infusion, levosimendan swiftly reaches therapeutic concentrations in the bloodstream. Steady-state conditions are established within 5 h after the initiation of continuous infusion. The rate of elimination of levosimendan from the body is about 3 mL/min/kg, while the duration of its action is approximately 1 h [27]. Levosimendan is primarily metabolized through conjugation pathways, and a small portion undergoes reduction in the intestine to form active metabolites (Figure 1). OR-1896 and OR-1855 are the main metabolites detected in the body, and their presence is associated with the extended hemodynamic effects of levosimendan [28]. A significant proportion of the drug undergoes extensive metabolic transformation, leading to over 95% of the administered dose being eliminated within seven days. The main route of elimination involves urine and faeces, where most levosimendan and its metabolites are excreted in a biologically inactive conjugated form. Notably, only minimal amounts of the original medicine are excreted unchanged in the urine [29,30].

## 3. Clinical Use of Levosimendan

### 3.1. Inotropy

**Figure 2:**
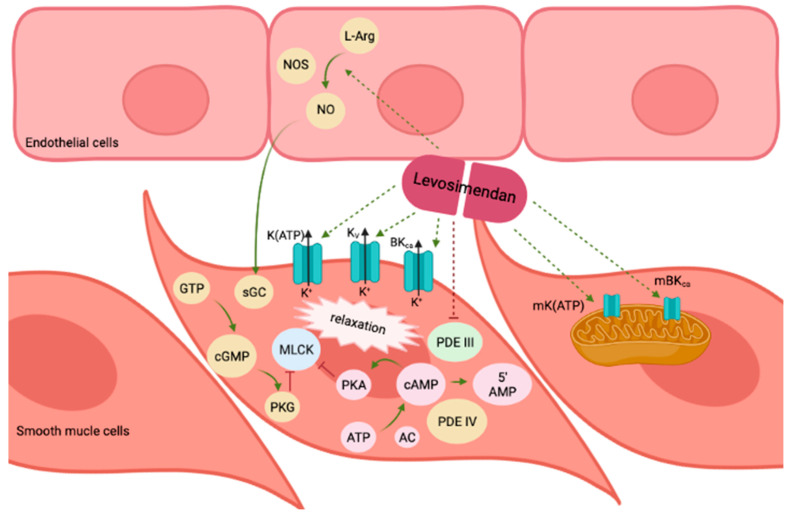
Schematic representation of the potential vasodilatory mechanisms triggered by levosimendan. Levosimendan demonstrates the ability to activate a range of mechanisms associated with vasodilation. Favourable impacts are represented by green arrows, while adverse effects are marked by red The distinctive effects of levosimendan are highlighted by dashed arrows. 5′AMP: 5′ adenosine monophosphate; AC, adenylate cyclase; ATP, adenosine triphosphate; cAMP, cyclic adenosine monophosphate; cGMP, cyclic guanosine monophosphate; GTP, guanosine triphosphate; l-Arg: l-arginine; MLCK, myosin light chain kinase; NO, nitric oxide; NOS, nitric oxide synthase; PDE III, phosphodiesterase III; PDE IV, phosphodiesterase IV; PKA: protein kinase A; PKG, protein kinase G; sGC, soluble guanylate cyclase.

By interacting with Ca^2+^-saturated cTnC within cardiomyocytes, levosimendan elicits a positive inotropic effect in cardiac muscle, as depicted in Figure 2. This effect is achieved without interrupting the relaxation process or triggering an elevation in oxygen consumption.

### 3.2. Vasodilation

Levosimendan, recognised as an inodilator, is often used to restore balance to the cardiovascular system in acute cardiac care. The vasodilatory effects of levosimendan result mainly from its ability to activate ATP-sensitive potassium K_ATP_ channels (Figure 2) [24,31,32]. The activation of these channels leads to the outflow of potassium ions, which causes vascular smooth muscle cells to hyperpolarize and subsequently relax. This relaxation results in vasodilation due to the closure of calcium channels [33].

One notable effect of levosimendan is its ability to significantly reduce pulmonary capillary wedge pressure (PCWP) and improve pulmonary circulation in cases of acute and advanced heart failure [34]. Preclinical investigations have suggested that levosimendan may relax pulmonary arteries by reducing the right ventricular afterload, and alleviate pulmonary edema by dilating pulmonary veins [35,36].

Levosimendan is a pharmaceutical substance that has been subject to extensive research into its vasodilatory impacts on various types of blood vessels. Initially, research efforts utilised rat mesenteric arterial myocytes and ventricular cardiomyocytes as in vitro test subjects, employing patch-clamp methodologies. These investigations revealed the activation of specific potassium channels, termed K_ATP_ channels [37,38]. Subsequent experiments in guinea pig hearts, rat arteries, mouse kidneys and human arteries reinforced these preliminary findings, establishing their applicability in ex vivo and in vivo contexts [39,40,41,42]. Interestingly, certain scenarios, such as in porcine coronary arteries and human umbilical arteries, revealed that the vasodilatory ramifications of levosimendan extended to the activation of alternative potassium channels (namely, K_V_ and BK_Ca_ channels) along with K_ATP_ channels. In human internal thoracic arteries, vasodilatation was attributed to the simultaneous involvement of K_ATP_ and BK_Ca_ channels [43,44,45]. Significantly, gender-specific distinctions emerged, as observed in the divergence between men and women in their responses to levosimendan; men showed a more pronounced vasodilation [46]. An in-depth analysis involving porcine coronary arteries revealed the involvement of intracellular elevation of cyclic adenosine monophosphate (cAMP) as a central factor in levosimendan-induced vasodilation. This cAMP elevation triggers protein kinase A activation while inhibiting myosin light chain kinase, ultimately leading to blood vessel relaxation [47]. The involvement of cAMP was not surprising, given levosimendan’s selective inhibition of phosphodiesterase III (PDE III) without affecting PDE IV at lower drug concentrations [48]. Additionally, research on porcine coronary endothelial cells suggested another vasodilatory mechanism involving nitric oxide (NO) and cyclic guanosine monophosphate (cGMP)-protein kinase G-myosin light chain kinase axis, further enhancing the vasorelaxation effects of levosimendan [49]. This interaction between K_ATP_ channel activation and NO signalling was also shown to reduce cell death in pigs when levosimendan was administered directly into the coronary arteries [50]. The long-acting derivative of levosimendan, known as OR-1896, also exhibited vasodilatory properties by inducing activation of BK_Ca_ channels in coronary microvessels and K_ATP_ channels in skeletal muscle arterioles in rat experiments [51]. In resistance arterioles in real physiological settings, both levosimendan and OR-1896 showed comparable efficacy in inducing vasodilation, predominantly attributed to the activation of K_ATP_ channels [52]. Furthermore, the vasodilatory effects generated by levosimendan were observed in an isolated human portal vein and human saphenous vein preparations. These responses were governed by the interaction of K_ATP_ and BK_Ca_ channels [53,54].

In summary, preclinical studies using different potassium channel blockers have indicated that levosimendan-induced vasodilation occurs through multiple mechanisms, which vary depending on the vascular bed. These findings shed light on the complex and versatile nature of levosimendan’s actions in the cardiovascular system (see Table 1).

### 3.3. Organ Protection

In addition to its favourable impact on myocardial contractility, levosimendan exhibits organ-protective properties by activating mitochondrial K_ATP_ channels (Figure 1). This mechanism contributes to the drug’s ability to safeguard organs from injury during ischemia–reperfusion, promoting better outcomes in conditions where organ damage is a concern [56].

Mitochondrial K_ATP_ channels play a vital role in maintaining cellular integrity by stabilizing mitochondrial metabolism during periods of reduced blood flow (ischemia) [57,58,59]. These organelles are involved in both apoptosis (programmed cell death) and necrotic cell death, making them central to cellular survival and damage processes. They are also implicated in the mechanism of preconditioning, where cells develop resistance to subsequent episodes of ischemia. Mitochondrial K_ATP_ channels orchestrate a number of vital functions including the regulation of mitochondrial matrix volume, matrix calcium levels, oxidative phosphorylation, nuclear calcium transients and the orchestration of gene expression [60]. Importantly, the occurrence of a specific channel, the mitochondrial permeability transition pore (mPTP) located in the inner mitochondrial membrane, is a key event in cell damage during episodes of ischaemic stress [61]. Prevention of the mPTP opening is the central mechanism to safeguard cardiac tissue during stress conditions [57,62,63]. Levosimendan has been shown to activate mitochondrial K_ATP_ channels, thereby inducing depolarisation in functional rat mitochondria [64]. This activation and subsequent stabilization of the organelle are postulated as the mechanism behind the protective effects of levosimendan on mitochondria, referred to as mitochondrial cardioprotection.

## 4. Repurposing Levosimendan for Oncology

### 4.1. Phosphodiesterase 3 Inhibition

Phosphodiesterase 3 (PDE3) belongs to the PDE enzyme family and consists of two subtypes, PDE3A and PDE3B. PDE3A is primarily found in the heart, platelets, vascular smooth muscle and oocytes. It plays significant roles in oocyte maturation and platelet aggregation processes. In contrast, PDE3B is predominantly expressed in white and brown adipose cells, hepatocytes, renal collecting duct epithelium and developing spermatocytes. This specific subtype plays a critical role in the intricate regulation of glucose and lipid metabolism [65].

The metastasis of cancer cells involves their migration into the surrounding extracellular matrix and adjacent blood vessels. Previous studies have demonstrated that increasing levels of cyclic adenosine monophosphate (cAMP) in cancer cells can inhibit their transcellular migration in vitro [66,67,68]. Therefore, drugs that effectively elevate cAMP levels in cancer cells have the potential to prevent metastasis in cancer patients.

Both levosimendan and its active metabolite, OR-1896, exhibit high selectivity as inhibitors of the phosphodiesterase (PDE) 3, which is responsible for breaking down cAMP [69].

Hao et al. suggest that the enzyme PDE3A plays a role in promoting the inflammatory nuclear factor NFκB signaling pathway by inhibiting the cAMP/PKA signaling pathway [70]. This inhibition leads to increased expression of the stem cell marker OCT4, which is associated with cancer stemness. Additionally, PDE3A facilitates the translocation of CCDC88A from the cytoplasm to the nuclei, contributing to the invasion–metastasis cascade in breast cancer [70]. In a study using human colon cancer cells (DLD-1), it was observed that cellular migration induced by soluble fibronectin or fetal bovine serum (FBS) was significantly reduced when treated with cilostazol, an inhibitor of PDE3. The migration suppression by cilostazol was approximately 92.3% for soluble fibronectin-induced migration and 84.6% for FBS-induced migration in control cells. Moreover, in a phagokinetic assay where cellular migration was assessed based on the phagocytosis track of gold particles stimulated by soluble fibronectin, cilostazol reduced cellular migration by approximately 67.3% in control cells. Additionally, in a transcellular migration assay, cilostazol effectively inhibited cancer cell invasion induced by FBS [71].

**Figure 3:**
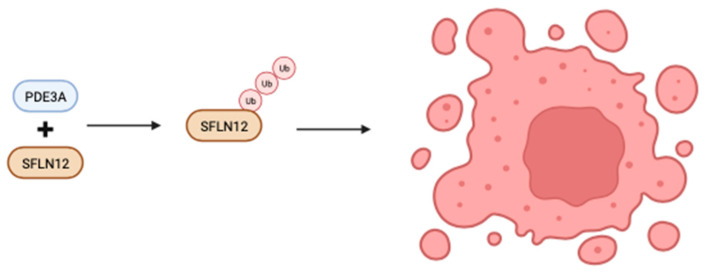
Inhibitors of PDE3A and PDE3B kills cancer cells by inducing PDE3A/B interactions with SFLN12.

In gastrointestinal stromal tumors, compared to other types of human cancers, PDE3A and PDE3B are highly expressed, offering a promising avenue for targeted therapy. DNMDP is a potent and selective inhibitor of PDE3A and PDE3B, effectively eliminating cancer cells by promoting interactions between PDE3A/B and SFLN12, a critical protein in this process (Figure 3). An analog of DNMDP, BRD9500, has demonstrated similar activity and shows promise in combating cancer in an SK-MEL-3 xenograft model. BRD9500 effectively inhibits PDE3A and PDE3B with IC50 values of 10 and 27 nM, respectively. Its administration through intravenous and oral routes in mice resulted in sustained high plasma levels over an eight-hour period, making it a valuable candidate for in vivo xenograft testing [72].

Collectively, these findings highlight the potential role of PDE3 as a promising therapeutic target for cancer. Targeting PDE3 using specific inhibitors such as levosimendan holds potential for inhibiting tumor growth and preventing metastatic spread in cancer patients.

### 4.2. Nitric Oxide Production

Levosimendan triggers nitric oxide (NO) production in endothelial cells through the activation of specific cellular pathways involving key proteins known as p38 mitogen-activated protein kinases (MAPKs), extracellular signal-regulated kinase (ERK) and protein kinase B (PKB/Akt) [49]. Nitric oxide is of significant importance due to its involvement in a number of vital biological activities. This highly dynamic molecule, characterised by its instability and fat-soluble nature, possesses the ability to easily permeate cell membranes by diffusion [73].

NO have dual effects in cancer: at a low concentration, similar to those generated by constitutive nitric oxide synthases (NOS), NO mainly acts as a signaling molecule by nitrosylating soluble guanylate cyclase (sGC) to increase cyclic guanosine monophosphate (cGMP) levels. This leads to vasodilation and also promotes angiogenesis and neovascularization through the activation of signaling pathways involving hypoxia-inducible factor 1 alpha (HIF1α) and vascular endothelial growth factor (VEGF) [74]. These effects can support tumor growth and spread. In certain cancers, upregulation of sGC has been associated with acquired chemotherapy resistance [75]. However, at a higher concentration, NO has been shown to inhibit cell proliferation and induce apoptosis leading to cancer cell death [76,77]. Indeed, the role of NO in cancer biology involves both pro-tumour and anti-tumour effects, which significantly influence cellular responses to various stressors. NO can affect multiple aspects of cancer development and progression, including DNA damage, oncogene activation, cell metabolism, DNA repair enzymes, tumour suppressor genes, apoptosis and metastasis [78].

Numerous investigations have proven the ability of NO to prevent the proliferation of various types of cancer cells, as evidenced by studies [79,80,81,82,83]. One particularly promising substance, known as GIT-27NO, serves as a source of NO and has demonstrated the ability to prevent the growth of prostate cancer cells (PC3 and LnCap) in a dose-dependent manner, as shown in experiments performed on mice without functional immune systems [84]. In addition, a different compound called Saquinavir, derived from the HIV protease inhibitor with NO properties, was observed to stimulate programmed cell death (apoptosis) and promote the synthesis of the pro-apoptotic cell death mediator BCL-2 interacting (Bim) in PC3 cells. In animal studies, Saquinavir with NO functionality (Saq-NO) exhibited greater inhibition of PC3 xenotransplants compared to the control [85].

**Figure 4:**
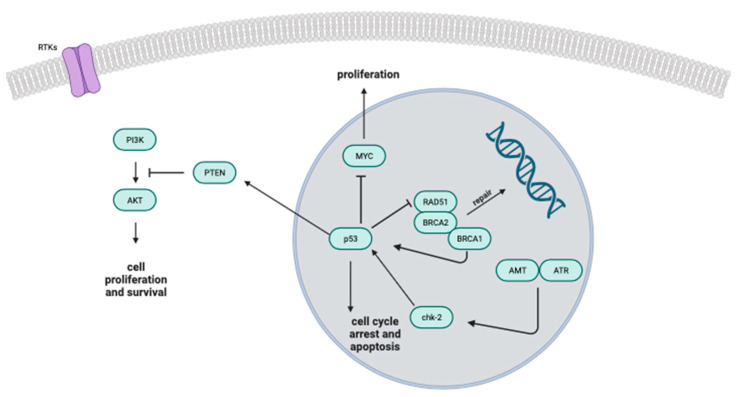
The diagram portrays the comprehensive signalling model involving tumour suppressors like p53 and BRCA1. It visually represents how various molecules, well-established for their roles in influencing cell proliferation, DNA damage response and cell cycle control interact within the regulatory pathway.

The potential anticancer influences attributed to NO are thought to materialise through the enhancement of a specific biological pathway encompassing BRCA1, Chk1, p53 and p21 (Figure 4). This intricate pathway holds a key responsibility in managing cell cycle progression, and possesses the ability to initiate cell cycle arrest as well as cell death when faced with a DNA deficiency or internal cellular stress [86].

Moreover, NO has been found to enhance the sensitivity of cancer cells to radiation, rendering them more susceptible to destruction. In the field of cancer radiotherapy, a notable obstacle is the presence of hypoxic cells in tumours characterised by their limited oxygen content, which makes them less responsive to radiation treatment. Remarkably, NO has demonstrated the ability to increase the responsiveness of these hypoxic cells to radiotherapy. This improvement is achieved through mechanisms that lead to an elevation of oxygen levels, achieved by influencing blood circulation and cellular oxygen uptake [87]. Furthermore, when NO collaborates with ionising radiation it triggers a synergistic effect, inducing apoptotic cell death through activation of the p53 pathway. Of note is a study that administered NO donors to colorectal cancer cells, resulting in a significant increase in the cells’ sensitivity to radiation treatment [88,89].

In summary, NO shows immense potential as a potent tool in cancer treatment, especially when used in conjunction with radiation therapy. By sensitizing hypoxic cells and triggering apoptotic cell death, NO has the capacity to enhance the efficacy of cancer treatment and yield better outcomes for patients.

### 4.3. Reactive Oxygen Species Reduction

Reactive oxygen species (ROS) are remarkably reactive molecules containing oxygen atoms that arise naturally as byproducts of cellular metabolism [90]. This category encompasses entities such as superoxide anion (O_2_^•−^), hydrogen peroxide (H_2_O_2_) and hydroxyl radical (^•^OH) [91]. Knowledge from in vitro and other model-based investigations has solidified the understanding that cancer cells possess high levels of ROS. These elevated concentrations of ROS play a key role in promoting and reconfiguring a diverse set of cellular processes. Specifically, they orchestrate signalling pathways linked to energy production, protective mechanisms, immune responses, resistance against harmful substances (xenobiotics), orchestration of programmed cell death (apoptosis) and cellular reactions in response to signals indicating cell death [92].

**Figure 5:**
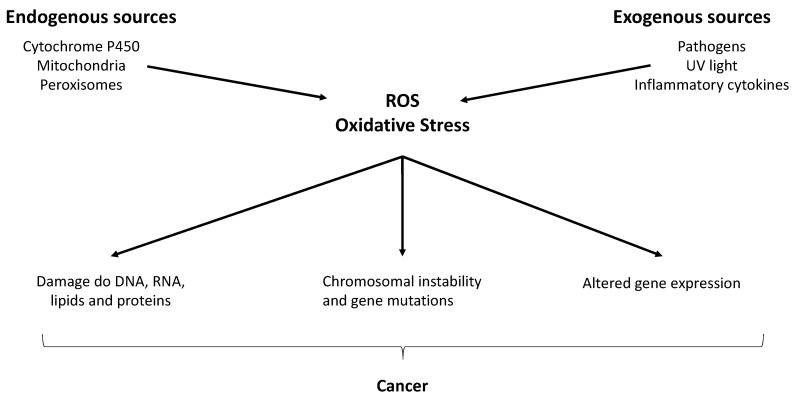
Pathways illustrating the sources of reactive oxygen species and its role in the development of cancer [99].

While ROS are produced in small amounts during normal cellular processes, excessive production or inadequate removal of ROS can lead to oxidative stress, which can damage cellular components such as proteins, lipids and DNA, and has been implicated in carcinogenesis (Figure 5) [93]. Elevated levels of ROS have been associated with cancer development and progression [94]. ROS can promote tumor initiation [95], growth [96], angiogenesis [97], invasion [98] and metastasis [94] through several mechanisms.

ROS have been extensively studied in the context of cancer and its various characteristics. They play a significant role in several key aspects of cancer development such as stemness and heterogeneity, suggesting that controlling ROS levels could be a promising approach to mitigating cancer progression. The connection between ROS and inflammation is well-established and is a crucial factor in the development of cancer [100]. When cancer cells experience different cellular events their ROS levels increase, leading to oxidative stress. As cancer cells navigate through various cellular events their ROS levels rise, triggering oxidative stress. This sequence triggers the activation of redox-sensitive transcription factors and growth regulatory proteins including NF-kB, AP-1, HIF-1A and NRF2. The activation of these transcription factors leads to disruptive signalling and altered expression of oxidative and inflammatory mediators that are integral to the genesis of cancer and the complexities of its treatment [101,102]. In addition, cancer cells exude a range of mediators including oxidative and inflammatory components, which orchestrate a reconfiguration of surrounding cells and tissues in the tumour microenvironment (TME). The TME, a composition of distinct immune cells (both innate and adoptive) and fibroblasts emerges as a key determinant in cancer proliferation, metastasis and clinical outcomes [103]. ROS emerge as essential orchestrators to drive signalling pathways related to EMT, metastasis and unfavourable clinical consequences [104,105]. Metabolic reprogramming represents an additional key facet of cancer pathogenesis as it perpetuates a continuous supply of energy for unrestrained growth, proliferation and maintenance of stem cell-like characteristics in cancer cells. Accumulated research over the past decades has revealed the substantial role of ROS in reshaping metabolic dynamics in several cancer categories, encompassing both lymphoid and myeloid cells [106,107,108,109].

Reducing ROS levels in cancer cells as a therapeutic approach might prevent oncogene activation. Kipka et al. suggest that the active metabolites of levosimendan may inhibit inflammatory ROS formation by preventing the activation of MAPK signaling [110]. In accordance, another study proposes that this drug significantly suppresses ROS production by the activation of potassium channels [111]. In this way, we demonstrate again the anticancer potential of the drug levosimendan, quenching ROS levels and consequently preventing oncogene activation.

#### Plasma Malondialdehyde

The measurement of plasma malondialdehyde (MDA) is a predominant biomarker for assessing oxidative stress levels in the body [112]. Oxidative stress materialises when there is a mismatch between the generation of reactive oxygen species (ROS) and the body’s ability to effectively neutralise them or repair the resulting deficiencies [113].

Several studies have reported elevated serum or plasma MDA levels in association with various types of cancers, including breast cancer [114,115], oral cancer [116], lung cancer [114,117] and colorectal cancer [118]. Increased MDA levels in these studies suggest higher oxidative stress and lipid peroxidation in cancer patients [119]. This can occur due to various factors including increased metabolic activity, chronic inflammation and the presence of tumor cells themselves. Cancer cells often exhibit higher levels of oxidative stress compared to normal cells due to their increased metabolic demands and altered redox balance [116].

Recent evidence indicates that levosimendan could reduce MDA levels [120,121,122]. These findings suggest that levosimendan could have anticancer properties.

### 4.4. Drug Combinations

Previously, we evaluated the combination of levosimendan with 5-fluorouracil (5-FU), an antineoplastic drug, and found that this drug could help increase the cytotoxicity of 5-FU, decreasing the viability of bladder cancer cells [20].

In the study, the authors propose an innovative drug repurposing approach using levosimendan in the UM-UC-5 neoplastic cell line. The primary objective of this combination approach is to capitalize on the safety and established antitumor activity of levosimendan in cancer cells. By pairing this well-established antineoplastic drug (5-FU) with a repurposed drug known for its acceptable toxicological profile, we aim to enhance the efficacy of 5-FU while simultaneously reducing its required therapeutic dosage.

This research findings revealed a remarkable synergistic effect when combining levosimendan with 5-FU in treating UM-UC-5 cancer cells. This combination demonstrated a significantly greater reduction in cell viability compared to using the repurposed drug individually. Particularly noteworthy was the combination of 100 μM of levosimendan with the IC50 value of 5-FU (13.41 μM), which resulted in an impressive 59% decrease in cell viability compared to treating with 100 μM of levosimendan alone [20].

These results underscore the potential of this drug combination as a promising therapeutic approach for bladder cancer treatment. The observed synergistic effect indicates that the interaction between levosimendan and 5-FU enhances their anticancer properties, leading to a more potent and effective treatment.

The significant reduction in cell viability achieved through this combination suggests that it could offer a more targeted and efficient treatment option for bladder cancer patients. By utilizing lower doses of each drug in combination, we can potentially reduce the risk of adverse side effects while maximizing therapeutic benefits. These findings are encouraging and shed light on the potential of repurposing levosimendan alongside 5-FU as a novel and impactful strategy in bladder cancer therapy. Further investigations and clinical studies are warranted to validate the effectiveness of this drug combination and explore its potential benefits for patients in a clinical setting. Ultimately, our research contributes to advancing the field of cancer therapeutics and offers hope for improved treatment outcomes for individuals with bladder cancer.

## 5. Challenges and Perspectives

Furthermore, the practice of repositioning drugs faces significant obstacles. For example, if initial clinical trials do not meet current regulatory standards, additional Phase I trials may be required to collect the necessary data. The most successful cases are often the result of a thorough understanding of the pharmaceutical pharmacology or clinical efficacy, achieved through a retrospective analysis of the original uses of the medicine. These repositioned medicines are usually accompanied by less robust intellectual property guarantees, which in turn decreases the potential return on investment for companies [123]. Conducting repurposing clinical trials requires substantial financial resources to meet safety standards, verify efficacy and navigate in the absence of patent protection and commercialisation hurdles. Despite their cost-effectiveness and short time requirements, financial support for repurposing methods has been inadequate, coupled with short patent duration and less than optimal return on investment. However, the landscape is evolving as advanced artificial intelligence offers new insights into the correlations between diseases and drugs, increasing the likelihood of successful clinical trials during the development process [1]. This progression promises to accelerate patient access to new medicines, presenting a ray of hope to promptly treat symptoms of rare diseases and potentially even save lives.

Most drug repositioning initiatives are based on encouraging clinical or epidemiological results or the identification of efficacy in live systems. Given the rapid advancement of computational methods and the exponential growth of data, the multifaceted benefits of drug repositioning are about to become increasingly evident [124].

In conclusion, this methodology not only addresses challenges but also paves the way for faster therapeutic advances, ultimately benefiting patients and offering solutions to pressing healthcare needs.

## 6. Conclusions

Drug repurposing, with levosimendan as a prime example, offers a promising and efficient approach to drug development for oncology. By leveraging the existing knowledge about approved drugs, researchers can save valuable time and resources, accelerating the implementation of new treatments and discovery of novel targets. Levosimendan, originally approved for heart failure, has demonstrated diverse pharmacodynamics including phosphodiesterase 3 inhibition, nitric oxide production and reduction of reactive oxygen species, making it an attractive candidate for repurposing in oncology. Its ability to inhibit cancer cell migration, sensitize hypoxic cells to radiation and reduce oxidative stress showcases its multi-faceted anticancer properties. Additionally, levosimendan has been investigated in combination with traditional anticancer agents like 5-fluorouracil, resulting in a synergistic effect that enhances the efficacy of the treatment. The success of repurposing levosimendan demonstrates the vast potential of this strategy in drug discovery. By building on the well-documented safety and efficacy profiles of approved drugs, researchers can develop innovative and cost-effective therapies for various diseases, including cancer. The exploration of levosimendan’s diverse mechanisms of action in oncology opens up new possibilities for targeted and efficient treatments, potentially leading to improved outcomes for cancer patients. While the preclinical evidence is promising, further clinical studies are necessary to validate the effectiveness and safety of levosimendan as an anticancer agent in humans. Continued research and development in drug repurposing hold significant promise in advancing cancer therapeutics and offering new clinical options for patients in need of effective and accessible treatments. Ultimately, the success of drug repurposing, exemplified by levosimendan, highlights the value of leveraging existing knowledge to drive innovation and improve patient care in oncology and beyond.

## Figures and Tables

**Table 1:** Vasodilatory mechanisms induced by levosimendan and OR-1896.

Drug	Effector	Vascular Bed (Species)	Reference
Levosimendan	K(ATP)	Mesenteric artery (rat)	[37]
		Coronary circulation (guinea pig)	[40]
		Renal circulation (mice)	[42]
		Internal thoracic artery (human)	[39]
		Skeletal muscle arteriole (rat)	[51]
		Portal vein (human)	[53]
Levosimendan/OR-1896	K(ATP)	Resistance arteriole	[52]
Levosimendan	KV + BK_Ca_	Coronary artery (pig)	[43]
		Umbilical cord artery (human)	[44]
OR-1896	BK_Ca_	Coronary arteriole (rat)	[51]
Levosimendan	K(ATP) + BK_Ca_	Internal thoracic artery (human)	[45]
		Saphenous vein (human)	[54]
Levosimendan	cAMP	Coronary artery (pig)	[47]
Levosimendan	K(ATP) + cAMP + cGMP	Pulmonary circulation (cat)	[55]
		Pulmonary artery (guinea pig)	[35]
Levosimendan	K(ATP) + BK_Ca_ + cAMP + cGMP	Pulmonary vein (guinea pig)	[35]
Levosimendan	K(ATP) + K_V_ + cAMP + cGMP	Pulmonary circulation (human)	[36]
Levosimendan	NO	Coronary endothelial cells (pig)	[49]

BK_Ca_, large conductance Ca^2+^-activated K^+^ channels; cAMP, cyclic adenosine monophosphate; cGMP, cyclic guanosine monophosphate; K(ATP), ATP-sensitive K^+^ channel; K_V_ channels, voltage-gated K^+^ channels; NO, nitric oxide.

## Data Availability

Not applicable.
